# Palm-based tocotrienol-rich fraction (TRF) supplementation modulates cardiac sod1 expression, fxr target gene expression, and tauro-conjugated bile acid levels in aleptinemic mice fed a high-fat diet

**DOI:** 10.1186/s12263-024-00742-9

**Published:** 2024-02-27

**Authors:** Nur Aliah Natasha Md Shahrulnizam, Mohd Danial Mohd Efendy Goon, Sharaniza Ab Rahim, Sook Weih Lew, Siti Hamimah Sheikh Abdul Kadir, Effendi Ibrahim

**Affiliations:** 1 Institute of Medical Molecular Biotechnology (IMMB), Faculty of Medicine, Universiti Teknologi MARA (UiTM), Cawangan Selangor, 47000 Sungai Buloh, Selangor, Malaysia; 2 Institute of Pathology, Laboratory and Forensic Medicine (I-PPerFoRM), Universiti Teknologi MARA (UiTM), Cawangan Selangor, 47000 Sungai Buloh, Selangor, Malaysia; 3 Department of Biochemistry and Molecular Medicine, Faculty of Medicine, Universiti Teknologi MARA (UiTM), Cawangan Selangor, 47000 Sungai Buloh, Selangor, Malaysia; 4 Department of Physiology, Faculty of Medicine, Universiti Teknologi MARA (UiTM), Cawangan Selangor, 47000 Sungai Buloh, Selangor, Malaysia; 5 Department of Pediatrics, Faculty of Medicine, Universiti Teknologi MARA (UiTM), Cawangan Selangor, 47000 Sungai Buloh, Selangor, Malaysia

**Keywords:** Tocotrienols, Farnesoid X receptor, Animal model, High-fat diet, Antioxidant

## Abstract

Tocotrienol-rich fraction (TRF) has been reported to protect the heart from oxidative stress-induced inflammation. It is, however, unclear whether the protective effects of TRF against oxidative stress involve the activation of farnesoid X receptor (*fxr*), a bile acid receptor, and the regulation of bile acid metabolites. In the current study, we investigated the effects of TRF supplementation on antioxidant activities, expression of *fxr* and its target genes in cardiac tissue, and serum untargeted metabolomics of high-fat diet-fed mice. Mice were divided into high-fat diet (HFD) with or without TRF supplementation (control) for 6 weeks. At the end of the intervention, body weight (BW), waist circumference (WC), and random blood glucose were measured. Heart tissues were collected, and the gene expression of *sod1*, *sod2*, *gpx*, and *fxr* and its target genes *shp* and *stat3* was determined. Serum was subjected to untargeted metabolomic analysis using UHPLC-Orbitrap. In comparison to the control, the WC of the TRF-treated group was higher (*p* >0.05) than that of the HFD-only group, in addition there was no significant difference in weight or random blood glucose level. Downregulation of *sod1*, *sod2*, and *gpx* expression was observed in TRF-treated mice; however, only *sod1* was significant when compared to the HFD only group. The expression of cardiac *shp* (fxr target gene) was significantly upregulated, but* stat3* was significantly downregulated in the TRF-treated group compared to the HFD-only group. Biochemical pathways found to be influenced by TRF supplementation include bile acid secretion, primary bile acid biosynthesis, and biotin and cholesterol metabolism. In conclusion, TRF supplementation in HFD-fed mice affects antioxidant activities, and more interestingly, TRF also acts as a signaling molecule that is possibly involved in several bile acid-related biochemical pathways accompanied by an increase in cardiac *fxr shp* expression. This study provides new insight into TRF in deregulating bile acid receptors and metabolites in high-fat diet-fed mice.

**Supplementary Information:**

The online version contains supplementary material available at 10.1186/s12263-024-00742-9.

## Introduction

Cardiovascular disease (CVD) is still considered as the major cause of death worldwide accounting for about 54% of all noncommunicable disease (NCD) mortality which is about 18 million of death in 2019 [1]. Effective preventive measures are crucial to reduce the number of deaths caused by CVD, this includes management and control of CVD risk factors. There are two classes of risk factors for CVD which is nonmodifiable and modifiable risk factors. Modifiable risk factors include behavioral, socioeconomic, psychosocial, and metabolic risk factors. Metabolic risk factors include raised blood pressure, overweight and obesity, hyperglycaemia (high blood glucose levels), and hyperlipidaemia (high levels of fat in the blood). Hyperlipidaemia with increased low-density lipoprotein (LDL), which has been reported as an atherogenic lipoprotein, possesses a strong CVD risk factor [2]. In laboratory setting, rats fed with a high-fat diet developed significantly greater adipose tissue, insulin resistance, and hyperleptinemia, which are associated with obesity [3–5] suggesting relationship between body fat and dietary fat although the exact mechanisms for the relationship are unclear. Several mechanisms have been proposed, and one possible mechanism is altered fat-induced satiation in response to prolonged fat ingestion and reduced sensitivity to cholecystokinin [6].

Farnesoid X receptor (FXR) is a nuclear receptor subfamily 1, group H, member 4 (NR1H4). It was first discovered in mice and rats as an orphan nuclear receptor [7, 8] and later is identified as a nuclear receptor for bile acid that is highly expressed in the liver, gastrointestinal tract, adipose tissue, pancreas, and kidney [9, 10]. It is known to act as a key metabolic regulator for the regulation of systemic energy. Activation of FXR induces its target gene, small heterodimer partner (SHP), which accounts for the inhibition of cholesterol 7α-hydroxylase (CYP7A1), phosphoenolpyruvate carboxykinase (PEPCK), and sterol regulatory element binding-protein 1c (SREBP-1c). Additionally, studies have shown that the induction of FXR plays significant roles in inflammatory pathways. Activation of FXR in immune cells inhibits tumor necrosis factor-α (TNF-α) production and suppresses nuclear factor kappa-light-chain-enhancer-of activated B cells (Nf-kB) and interferon gamma (IFN-γ)-related genes in macrophages. The inflammatory mediators of inducible nitric oxide synthase (iNOS), cyclooxygenase-2 (COX-2) and interferon-γ-inducible protein 10 (IP-10) induced by administration of lipopolysaccharides are repressed following FXR receptor activation [11]. FXR and its target genes, SHP, and phospholipid transfer protein (PLTP) were first discovered in the cardiovascular system in 2004 [12]. It was present in the normal vascular smooth muscle of the coronary artery, aorta, cardiac muscle and diseased hypertrophic heart, heart failure, and myocardial infarction. It was also expressed in neonatal rat ventricular myocytes, H9c2 cardiac cells, and neonatal rat cardiac fibroblasts [13]. FXR expression was reported in the whole heart and cardiac vessels of obese fa/fa Zucker rats and neonatal cardiomyocytes isolated from Wistar rats [14].

The palm-based tocotrienol-rich fraction (TRF) is a vitamin E mixture that consists of 25% α-tocopherol and 75% tocotrienols. Both tocopherol (T) and tocotrienol (T3) consist of four isomers: alpha (α), beta (β), gamma (γ), and delta (δ) [15]. Tocotrienol supplementation has been proven to prevent the development of atherosclerosis in studies using animal models [16] and is evident in human studies [17] In an earlier study, heterozygous apoE knockout mice fed an atherogenic diet supplemented with a vitamin E mixture derived from palm oil observed a substantial decrease in atherosclerotic lesion formation [18]. Recently, a study has reported that low-dose supplementation with TRF causes decreased endothelial activation and inflammation and reduced atherosclerotic lesions in the aorta of rabbits with induced atherosclerosis [19]. Similar findings were observed in apolipoprotein E (apoE^−/−^) knockout mice fed an atherogenic diet following TRF supplementation [20]. In addition, a study showed that palm oil tocotrienol-rich extract was able to restore the endothelial function of aortic rats in the presence of oxidative stress by scavenging superoxide radicals produced by hypoxanthine/xanthine oxidase [21], while another study demonstrated that pre- and post-treatment with palm TRF significantly increased neonatal rat cardiomyocyte cell viability after exposure to H_2_O_2_ [22].

Numerous studies have demonstrated the cardioprotective roles of TRF such as inhibition of inflammation [23] and reduced development of atherosclerosis [24]. However, the roles of FXR in mediating the effects of TRF remain unclear, especially in the cardiac tissue of mice subjected to an increased risk of CVD. Therefore, in the present study, the effects of TRF supplementation on the cardiac function of aleptinemic mice subjected to HFD were investigated in terms of body weight, waist circumference, random blood glucose, and antioxidant activities comprising *superoxide dismutase 1* (*sod1*), *superoxide dismutase 2* (*sod2*), and *glutathione peroxidase 1* (*gpx1*) gene expression. In addition, the expression of cardiac *fxr* and its target genes *shp* and *signal transducer and activator of transcription 3* (*stat3*) were also measured. Finally, serum untargeted metabolomics was assessed and compared between the TRF-supplemented group and the control group. Determining the gene expression and metabolites altered by TRF supplementation provides insight of the role and mechanism of action of TRF supplementation on the cardiac tissue of mice with an increased risk of CVD.

## Methods

### Animals and reagents

Fourteen 6-week-old male mice B6.Cg-LepOb/J strain (leptin-deficient mice) were purchased from Jackson Laboratory (Maine, USA). Mice were housed individually under controlled temperature (23 ± 1 °C) and humidity of 50 ± 5% under a strict 12:12 light/dark cycle. Mice were provided with food and reverse osmosis water ad libitum. After 2 weeks of acclimatization, mice were randomly divided into two groups (*n* = 7 per group). Throughout this study, both groups were fed a high-fat diet (HFD; Altromin, Germany) consisting of 60% fat, 20% carbohydrates, and 20% protein. One group was fed a high-fat diet without any intervention (HFD group). Another group was supplemented with tocotrienol-rich fraction (TRF) at 200 mg/kg/day on top of their HFD for 6 weeks. During the study, the body weights of the mice were measured weekly at the beginning of every week, while the waist circumference was measured once at the end of the study. Random blood glucose levels of the mice were also measured at the end of the study, using a digital glucometer (Accu-cek, Roche Diagnostic, Switzerland) before being euthanized with rapid cervical dislocation. Blood was collected by cardiac aspiration using a 26-G needle (Terumo, Japan) and placed in a plain blood tube (red-top tube; Becton Dickinson, USA). The blood was settled at room temperature for 20 min before being centrifuged at 1500 g and 4 °C for 10 min. The supernatant formed was transferred into microtubes and stored at − 80 °C for metabolomics analysis. Cardiac tissue from every mouse was dissected out by incising the ascending aorta, cleaned using phosphate-buffered saline (PBS), and snap-frozen using liquid nitrogen. Frozen cardiac tissue was stored at − 80 °C until further analysis. All procedures performed in mice were approved by the Universiti Teknologi MARA (UiTM) Committee on Animal Research and Ethics. The study was conducted at the Laboratory of Animal Care Unit, Faculty of Medicine, UiTM Sungai Buloh Campus, Selangor, Malaysia. Unless otherwise specified, all reagents are purchased from Sigma-Aldrich.

### Cardiac RNA extraction, cDNA synthesis and RT-qPCR

Total RNA was extracted from cardiac tissues from both groups using the GeneJET RNA Purification Kit (Thermo Scientific, USA). Each cardiac tissue sample weighing 30 mg was homogenized in a 1.5-ml centrifuge tube containing 300 µL of lysis buffer supplemented with 14.3-M β-mercaptoethanol using a rotor–stator homogenizer for 40 s. Briefly, all extractions were performed according to the manufacturer’s protocol to collect purified RNA in the final step. The concentration of RNA was measured and recorded using a NanoDrop. RNA was stored at − 80 °C for further use. The extracted total RNA (20 ng) was reverse transcribed into complementary DNA (cDNA) using a Maxima First-Strand cDNA Synthesis Kit (Thermo Scientific, USA). A reaction mixture was prepared by combining all components for the RT reaction into a sterile, RNase-free tube as indicated in Table S1. The reaction mixture was mixed gently and centrifuged before being incubated for 10 min at 25 °C followed by 15 min at 50 °C. The reaction was terminated by heating at 85 °C for 5 min. Subsequently, RT-qPCR was carried out using Maxima SYBR Green qPCR Master Mix (Thermo Scientific, USA). Sense and antisense primers were designed using Primer Premier software ( Table S2). Housekeeping genes comprised *rpl4, β-actin* and *gapdh*. Target genes comprised of *sod1*, *sod2*, *gpx1*, *fxr*, *shp*, and *stat3*. First, Maxima SYBR Green qPCR Master Mix components were thawed, vortexed gently, and briefly centrifuged. Then, the reaction master mix ( Table S3) was prepared by adding all components (except template cDNA) for each 25-µL reaction to a tube at room temperature. Template cDNA was added (≤ 500 ng) to the individual PCR tubes containing the master mix. The reactions were gently mixed without creating bubbles. The CFX96 Bio-Rad Thermal Cycler was programmed according to the manufacturer’s recommendations ( Table S4), and PCR tubes were placed in the real-time cycler to initiate the cycling program.

### Untargeted metabolomics analysis by UHPLC‒MS

All sera collected by cardiac aspiration were thawed prior to preparation. Once thawed, 100 µL of each serum sample was individually transferred into sterile 1.5-mL microtubes on ice. A total of seven sera from the TRF group (*n* = 7) and five sera from the HFD group (*n* = 5) were prepared for untargeted metabolomics analysis. The sera were mixed with 300 µL of methanol (Optima® LC/MS, Fisher Chemical, USA) for deproteination. The mixture was vortexed for 15 s and centrifuged for 15 min at 15,800 G and 4 °C. The formed supernatant was carefully pipetted and transferred into a sterile 2-mL microtube. Finally, the supernatant was dried using a concentrator (Concentrator plus, Eppendorf, Germany) in VAQ mode for 4 h. The dried samples were reconstituted by ultrahigh-performance liquid chromatography‒mass spectrometry (UHPLC‒MS) untargeted metabolomics analysis with 100 µL of LCMS-grade water (W6-4 Water, Optima® LC/MS, Fisher Chemical, USA), vortexed for 15 s and transferred into glass vials by filtering with a 0.22-µm cellulose membrane filter. Triplicates of blank samples were prepared by pipetting and filtering 200 µL of LCMS-grade water. All prepared samples and blanks were analyzed using UHPLC (UltiMate™ 3000, Thermo Scientific™, USA) and MS (Q Exactive HF Orbitrap-MS, Thermo Fisher Scientific, USA). LCMS-grade water with 0.1% formic acid was used as mobile phase A, and acetonitrile (ACN) with 0.1% formic acid was used as mobile phase B. The UHPLC was equipped with a C18 column (100 mm × 2.1 mm, 1.7 µm; Synchronis™, Thermo Scientific™, USA). Chromatographic separation was carried out at a flow rate of 450 µL/min. The column temperature was set to 55 °C and 2 µL per injection. The elution gradient was performed as outlined in Table S5. Mass spectrometry (Q Exactive HF Orbitrap-MS, Thermo Fisher Scientific, USA) scanning was conducted at 50 arbitrary unit (AU) sheath gas flow rate (GFR), 18 AU auxiliary GFR, 0 AU sweep GFR, 55 AU S-lens, capillary temperature at 320 °C, and auxiliary gas heater temperature at 300 °C. Electron-spray ionization was performed in positive and negative mode. In positive mode, the spray voltage was set at 3.5 kV, while 3.0 kV of spray voltage was set for negative mode. A resolution of 60,000 with a scan range of 100–1000 (m/z) was set for MS scanning followed by MS/MS scans at a resolution of 15,000 with stepped normalized collision energies of 20, 40, and 60 AU. The generated spectra were preprocessed by Xcalibur™ version 3.1 (Thermo Fisher Scientific, USA).

### Metabolite features annotation

Prior to annotation of metabolites of the generated spectra from each sample, statistical analysis was carried out on both the HFD and TRF groups to identify metabolites that were significantly important. Data normalization, statistical analysis, and chemometric and univariate analyses were performed using MetaboAnalyst [25]. Chemometric analysis using principal component analysis (PCA) was carried out to project the PCA score of variation between TRF and HFD and plot. Univariate analysis using a volcano plot where fold-change above 1.5 and *p*-value less than 0.05 (*p* < 0.05) were set as the parameters when generating the plot. Using these parameters, a list of metabolite features in the TRF group against the HFD group that were significant with a fold-change above 1.5 was generated. The metabolite features were annotated using the m/z cloud, ChemSpider, CEU Mass Mediator (CEUMM), Human Metabolome Database, and METLIN.

### Statistical analysis

Body weight, waist circumference, random blood glucose, and the levels of gene expression were determined and compared by unpaired Student’s *t*-test using SPSS. A *p* < 0.05 was considered statistically significant. Unless otherwise specified, all the data are presented as mean ± SEM.

## Results

### Effect of tocotrienol-rich fraction (TRF) supplementation on body weight, waist circumference and random blood glucose of high-fat diet mice

**Fig. 1:**
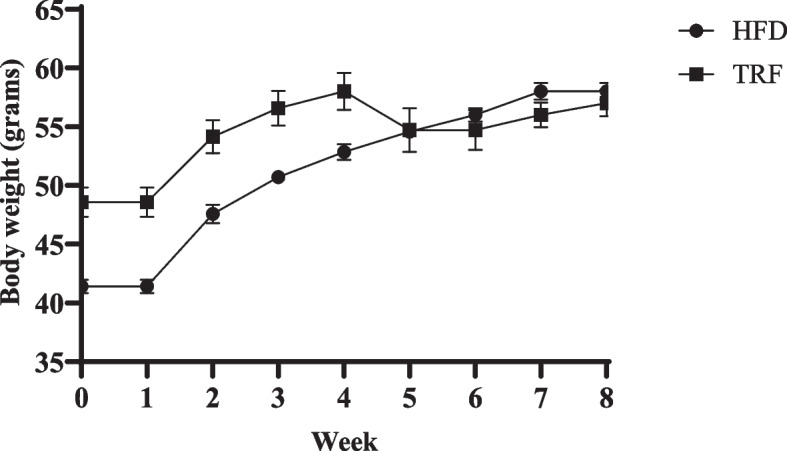
Changes in mouse body weight for 8 weeks of HFD. Mice fed a HFD supplemented with TRF showed a decrease in body weight after 5 weeks of supplementation

**Fig. 2:**
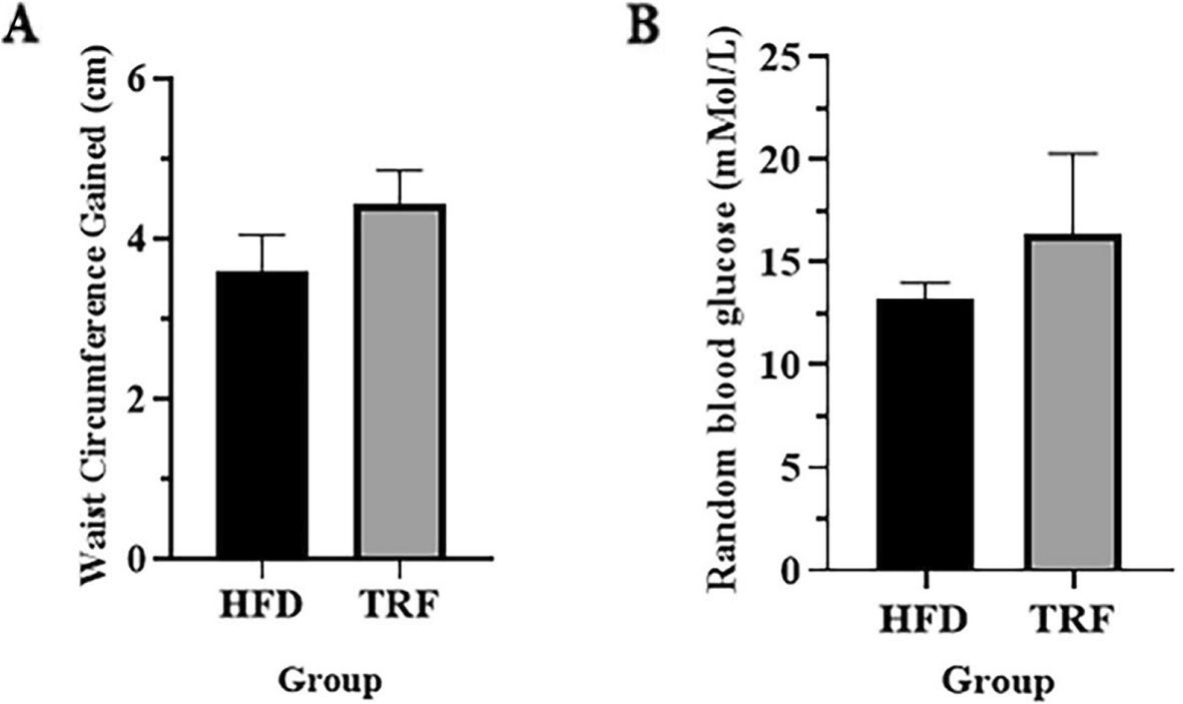
Comparison of waist circumference gained (**A**) and random blood glucose (**B**) at the end of the study between the TRF and HFD groups. The waist circumference of mice supplemented with TRF was found to be higher than that of the HFD group at the end of the study (**A**). The waist circumference measured was found not significant between TRF group with the HFD group (*p* > 0.05). The random blood glucose level measured in the TRF group (16.40 ± 3.89) was higher than that in the HFD group (13.24 ± 0.74) (**B**). The mean difference was not significant (*p* > 0.05). Values are expressed as the mean ± SEM

Mice were fed a high-fat diet (HFD) for 8 weeks. The body weight of all mice gradually increased over time. Feeding with HFD increased body weight; however, supplementation with TRF while on HFD limited the body weight gain after week 4 (Fig. 1). However, the difference in body weight recorded at the end of the study between the groups was not significant (*p* > 0.05). At the end of the study, the total waist circumference (WC) gained at the of week 8 for the HFD group was 3.57 cm ± 0.18, while that of the TRF-supplemented group was 4.42 cm ± 0.16 (Fig. 2A). The WC measured was not significantly different between TRF group with the HFD group (*p* > 0.05). The mean random blood glucose of the high-fat diet-fed mice supplemented with TRF in comparison with the HFD group is shown in Fig. 2B. The random blood glucose levels measured in the HFD and TRF groups were 13.24 mMol/L ± 0.74 and 16.40 mMol/L ± 3.89, respectively, with no significant difference (*p* > 0.05).

### Effects of tocotrienol-rich fraction (TRF) supplementation in the heart of high-fat diet-fed mice on superoxide dismutase (sod1 and sod2) and glutathione peroxidase (gpx1) gene expression

**Fig. 3:**
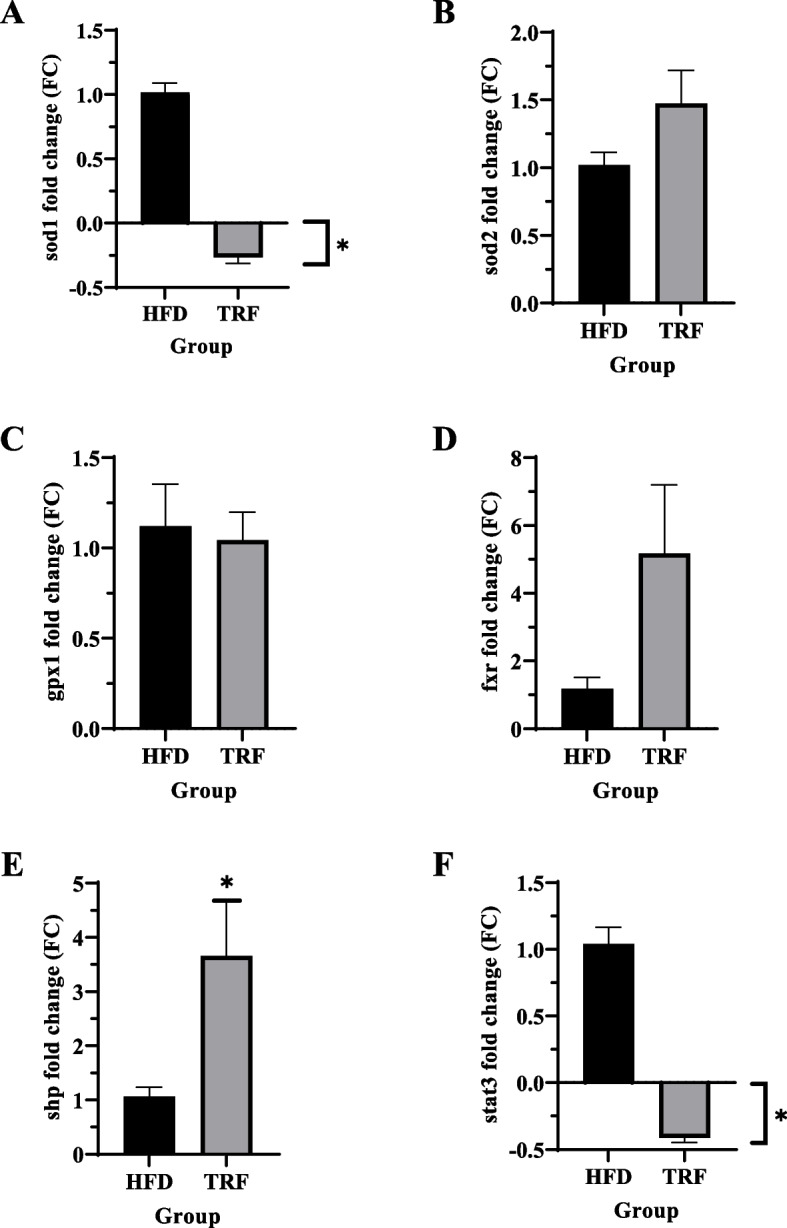
Fold-change of cardiac gene expression of sod1 (**A**), sod2 (**B**), gpx1 (**C**), fxr (**D**), shp (**E**), and stat3 (**F**) in TRF group compared to HFD group. The gene expression of sod1 and stat3 was found to be significantly downregulated in the TRF group (*p < 0.05). Other genes, such as sod2, gpx1, and fxr, were upregulated but not significantly (*p* > 0.05) except for shp (*p < 0.05). Values are expressed as the mean fold-change (FC) with standard error of the mean (SEM)

There was a significant downregulation of *sod1* expression with TRF supplementation (0.27-fold lower) compared to the HFD group (*p* < 0.05) (Fig. 3A). Meanwhile, *Sod2* expression was 1.48-fold higher in TRF mice (Fig. 3B), but not statistically significant compared to the HFD group. Relative to the HFD group, downregulation (1.04-fold) in gpx1 expression was observed in the TRF group but was not significant (*p* > 0.05; Fig. 3C).

### Effects of tocotrienol-rich fraction supplementation on the fxr and its target genes (shp and stat3) expression in the heart of high-fat diet-fed mice

The expression of *fxr* was upregulated in the heart of high-fat diet-fed mice supplemented with TRF (5.17-fold) compared to the HFD group, but the difference was not significant (Fig. 3D). The significant upregulation of heart expression of shp, a fxr target gene, was observed in the TRF group (3.66-fold) compared to the HFD group (*p* ≤ 0.05; Fig. 3E). A significant downregulation of stat3 was observed in the TRF group (0.41-fold) compared to the HFD group (*p* < 0.05; Fig. 3F).

### Principal component analysis (PCA) of TRF group against HFD group

**Fig. 4:**
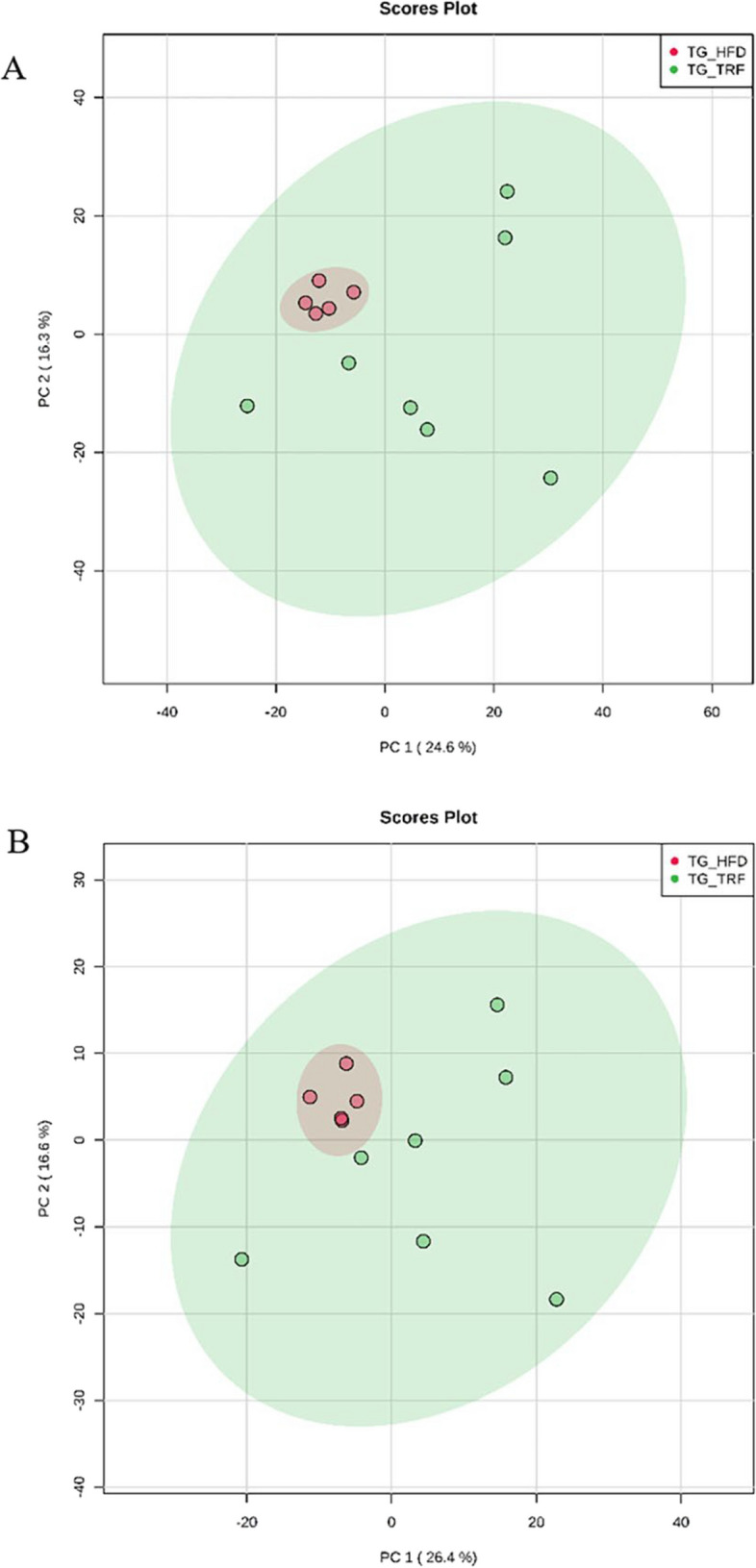
Principal component analysis (PCA) plot of the TRF group (  ) against the HFD group (  ) in positive mode (**A**) and negative mode (**B**). The PCA score in positive mode showed a total variation of 40.9% and 43% in negative mode

A total of 31,248 peaks were generated in UHPLC‒MS-positive mode when comparing the TRF group against the HFD group. From the total of seven samples per group, the average total peaks detected in each sample was 2604, which gave rise to 2060 peak groups. The PCA score generated a total variation of 40.9%, whereby its principal component 1 (PC1) score was 24.6%, while PC2 showed 16.3% variation in the TRF group when compared against the HFD group (Fig. 4A). In negative mode analysis, 15,204 number of peaks were detected. The average number of peaks per sample was 1267. This generated a total peak group of 1001. PCA showed a total variation of 43%, where the score on PC1 was 26.4% and that on PC2 was 16.6% (Fig. 4B). Further analysis using univariate analysis and volcano plots identified a total of 141 metabolic features when comparing the TRF group against the HFD group in positive mode analysis and 55 metabolic features in negative mode analysis. PCA score plot generated showed better separation between groups in HFD aleptinemia mice in comparison with wild-type mice (Figure S1). Therefore, metabolite profiling was carried out on aleptinemia mice only.

### Annotation of metabolic features in TRF group against HFD group

A total of 42 metabolites were successfully annotated from 196 metabolic features (Table S6). The metabolites comprised bile acids, lipids, sphingosine, and alkaloids. Metabolites such as isovaltrate (7.64-fold higher), ( +)-abscisic acid beta-D-glucopyranosyl ester (5.98-fold higher), and paucin (5.19-fold higher) were found to be the top three metabolites upregulated in the TRF group compared to the HFD group. N-Undecanoylglycine (6.19-fold lower), phytosphingosine (4.92-fold lower), and 2-amino-hexadecanoic acid (2.78-fold lower) were found to be the top three metabolites that were most downregulated in the TRF group compared with the HFD group.

### Joint-pathway analysis of annotated metabolites and gene expression (sod1, sod2, gpx1, fxr, shp, and stat3) in the TRF group

**Fig. 5:**
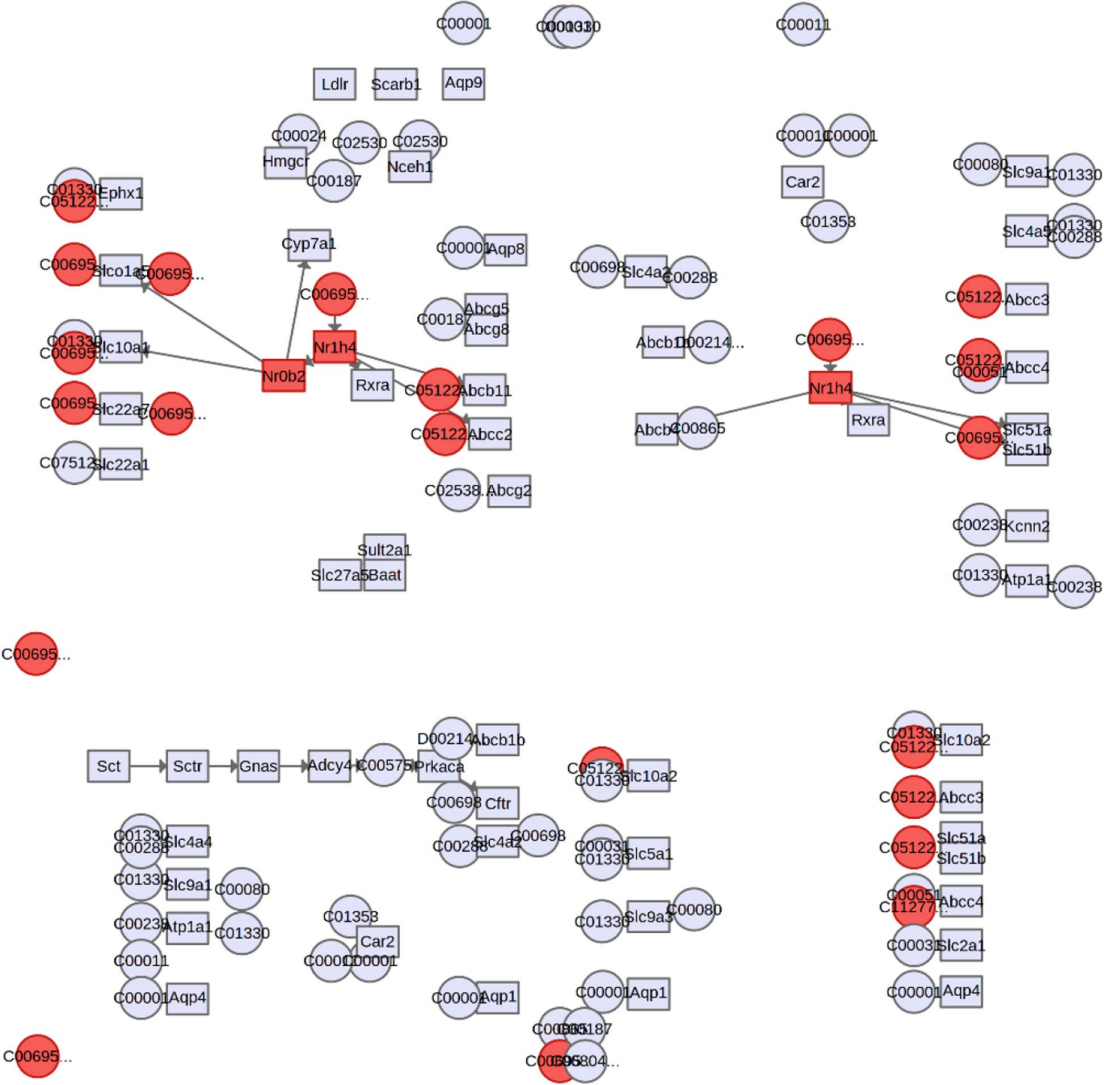
Bile secretion pathway possibly modulated by TRF supplementation in HFD-fed mice (*p* < 0.0001). Hit features are shown in red. Nr1h4, farnesoid X receptor (FXR); Nr0b2, small heterodimer protein (SHP); C05122, taurocholic acid; C00695, cholic acid

Joint-pathway analysis from the list of annotated metabolites and genes generated a total of 12 possible biochemical pathways that were influenced by TRF supplementation in mice fed a HFD (Table S7). The most likely and significant biochemical pathway modulated by TRF was found to be bile secretion (*p* < 0.0001), where fxr (mmu: 20186) and shp (mmu: 23957) and metabolites composed of taurocholic acid (cpd:C05122), taurochenodeoxycholic acid (cpd:C05465), and ouabain (cpd:C01443) were involved in the biochemical pathway (Fig. 5). Similarly, primary bile acid biosynthesis was also found to be a significant biochemical pathway involved (*p* < 0.05). The hit features were taurochenodeoxycholic acid (cpd:C05465) and taurocholic acid (cpd:C05122). Another significant biochemical pathway involved was biotin metabolism, where L-lysine (cpd:C00047) and biotin sulfone (cpd:C20387) were found to be involved. Apart from that, cholesterol metabolism was also found to be a significant biochemical pathway involved (*p* < 0.05), in which taurocholic acid (cpd:C05122) and taurochenodeoxycholic acid (cpd:C05465) were found to be involved.

## Discussion

This study investigates the effects of tocotrienol-rich fraction (TRF) supplementation in aleptinemic mice that were fed a high-fat diet (HFD) for 8 weeks. Feeding a HFD increases the risk of cardiovascular diseases by promoting cardiac injury, dyslipidemia, and oxidative stress [26, 27]. Mice that were subjected to HFD had an excessive increase in weight [28] and increased random blood glucose levels [29], both of which are risk factors for CVD. In this study, parameters of mice comprising body weight, random blood glucose, and waist circumference were measured, while the effects of TRF supplementation on mice fed a HFD on genes related to antioxidants, including *sod1*, *sod2*, and *gpx1*, were investigated in cardiac tissues. Furthermore, the role of the cardiac *fxr*, *shp*, and *stat3* genes was assessed by measuring their level of expression in TRF-supplemented mice. Finally, systemic metabolomics alterations were analyzed to provide insights into the metabolites that were most likely modulated or altered with TRF supplementation.

Mice subjected to HFD have been well reported to develop large body weight and increased blood glucose levels due to the consistent supply of surplus daily calories [30]. Although caloric restriction has been shown to control increases in body weight and improve blood glucose levels [31], TRF supplementation has also demonstrated similar effects on body weight [32] and blood glucose levels [33]. In the present study, the body weight of mice was shown to decrease after 4 weeks of TRF supplementation. However, the body weight in this group exhibited an upwards trend after week 5, which may be contributed by the excessive calories delivered by HFD, overcoming the effect of TRF. The δ-tocotrienol, as the major component of TRF, has been previously reported not able to significantly reduce body weight however promote smaller adipose tissue formation [34], and this possibly explain our finding where TRF supplementation did not affect body weight and waist circumference in comparison with HFD group. Therefore, we think that TRF supplementation under caloric restriction would promote promising properties by delivering optimal weight reduction in mice subjected to prior HFD feeding, which would further decrease the risk of CVD.

Apart from the positive outcome on body weight, TRF has been reported to improve fasting blood glucose (FBG) [35]. In this study, random blood glucose (RBG) was measured instead of FBG due to limitations in this study, where blood sera were dedicated for untargeted metabolomics analysis. The RBG level was higher in the TRF group than in the HFD group. A previous study demonstrated that TRF primarily exhibited antioxidant properties rather than exerting hypoglycemic effects in diabetic animals [36]. The present study recorded higher cardiac *sod2* expression in the TRF group than in the HFD group, which suggests increased antioxidant activity in the cardiac tissue of mice supplemented with TRF. Similarly, a study using an in vitro model demonstrated a higher *sod2* expression when supplemented with TRF [37]. Increased *sod2* activities with TRF supplementation suggest improved oxidative stress in mitochondria, as the gene is highly expressed in the organelle [38]. The antioxidant activities exerted by TRF were most likely limited to *sod2*, as *sod1* was found to be downregulated and *gpx1* was found to be only slightly decreased. Downregulation of *sod1* following TRF supplementation was also reported in other studies [39, 40], suggesting that *sod1* is most likely not modulated by TRF in promoting minimal *gpx1* activity. However, the positive effect of TRF supplementation was observed to be not limited to *sod2*, but it may act as a signaling molecule that affects several biochemical pathways based on findings in this study.

Joint pathway analysis combining the fold changes of investigated genes (*sod1*, *sod2*, *gpx1*, *fxr*, *shp*, and *stat3*) and metabolites as listed in Table S7 generated a total of 12 important biochemical pathways. The most significant biochemical pathway (*p* < 0.001) with a 4.39-fold higher fold change compared to the HFD group was bile secretion (Fig. 5). The genes involved in bile secretion with TRF supplementation were *fxr* (5.17-fold higher in TRF group) and *shp* (3.66-fold higher), and the metabolites involved were taurocholic acid (fold change: 3.28) and taurochenodeoxycholic acid (fold-change: 3.84). The increase in *fxr* gene expression, most likely from TRF supplementation, promotes the expression of *shp* as a result of nuclear translocation [41]. The increase in *shp* promotes the inhibition of bile acid reuptake by suppressing the Na^+^ taurocholate cotransporting polypeptide (NTCP) transporter. Compared to other target organs, the action of bile acids (BAs) on cardiomyocytes is indirect [42] in regulating myocardial function, and the expression of *fxr* has been reported to be lower than that in vascular smooth muscles [12]. However, other conjugated bile acids, such as taurocholic acid (TCA) and taurochenodeoxycholic acid (TCDCA), were found to be increased above threefold in the TRF group compared to the HFD group. The presence and increase in these bile acids (BAs), apart from being involved in BA secretion and primary BA synthesis (2.32-fold increase), were most likely suggestive of anti-inflammatory properties exerted by these BAs [43, 44] on the mice. Therefore, TRF supplementation in mice subjected to HFD was most likely elicited as a signaling molecule on *fxr*, thus modulating *shp* and *stat3* gene expression.

## Conclusion

In conclusion, TRF supplementation at 200 mg/kg/day for 8 weeks does not affect the body weight, blood pressure, or random blood glucose of HFD-fed mice. However, the expression of *sod1* was reduced significantly in TRF-supplemented mice, reflecting the role of TRF as an exogenous antioxidative substance. According to the metabolomic analysis, supplementation of TRF in HFD-fed mice may also act as a signaling molecule affecting several biochemical pathways, such as bile acid biosynthesis and secretion. Although the plasma level of bile acids was not measured in this study, the significant increase in fxr target gene *shp *and decrease in *stat3* following TRF supplementation suggests an interesting involvement of TRF in bile acid signaling.

### Supplementary Information

**Supplementary Material 1.**

**Supplementary Material 2.**

## Data Availability

No datasets were generated or analysed during the current study.
