# Supplementary material for: Palm-based tocotrienol-rich fraction (TRF) supplementation modulates cardiac sod1 expression, fxr target gene expression, and tauro-conjugated bile acid levels in aleptinemic mice fed a high-fat diet
Source: Genes Nutr. 2024 Feb 27;19:3. doi: 10.1186/s12263-024-00742-9 (PMC10898183; doi:10.1186/s12263-024-00742-9)
Supplement: Supplementary file 1 — Supplementary Material 1. [file 12263_2024_742_MOESM1_ESM.docx]

**Supplimentary (Figure)**


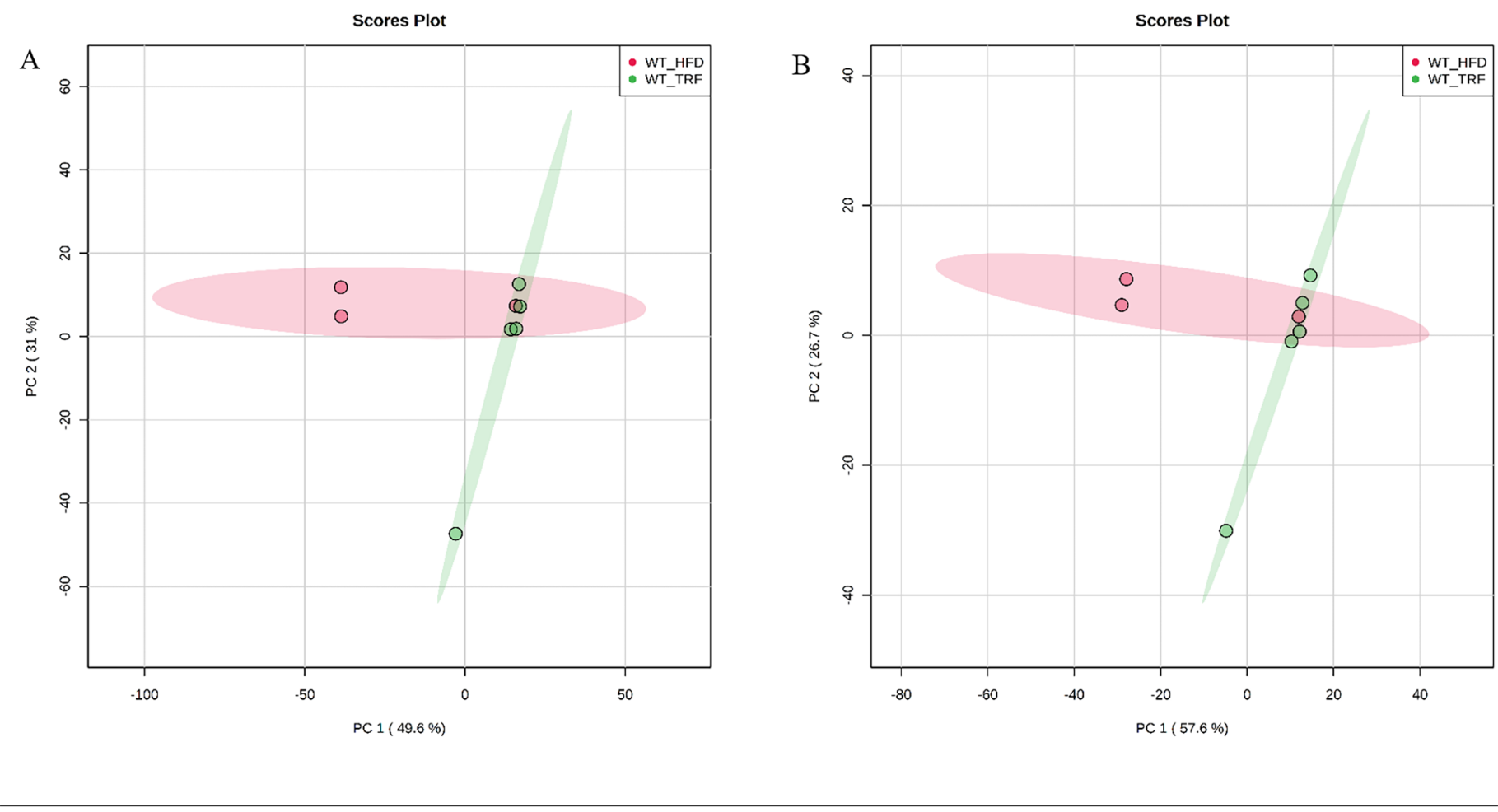


Figure S1: Principal Component Analysis (PCA) Score Plot of wild type mice with HFD only (I-HFD) and and HFD with TRF (TRF-HFD).
Plots represent positive mode (A) and negative mode (B). Both mode showed no clear separation between the groups.
I-TRF: ⚫, WT_TRF, n=5; I-HFD: ⚫, WT_HFD, n=3.
