# Supplementary material for: Palm-based tocotrienol-rich fraction (TRF) supplementation modulates cardiac sod1 expression, fxr target gene expression, and tauro-conjugated bile acid levels in aleptinemic mice fed a high-fat diet
Source: Genes Nutr. 2024 Feb 27;19:3. doi: 10.1186/s12263-024-00742-9 (PMC10898183; doi:10.1186/s12263-024-00742-9)
Supplement: Supplementary file 2 — Supplementary Material 2. [file 12263_2024_742_MOESM2_ESM.docx]

**Supplimentary (Table)**

Table S1: Reaction mixture for RT‒qPCR of extracted total RNA.

| **Component** | **Volume/reaction** |
| --- | --- |
| 5X Reaction Mix | 4 µL |
| Maxima Enzyme Mix | 2 µL |
| Template RNA | 1 pg – 5 µg |
| Water, nuclease-free | to 20 µL |
| Total Volume | 20 µL |

Table S2: Primer sequences

| Genes | NCBI Accession Number | Sequence |
| --- | --- | --- |
| fxr | NM_009108 | Forward: 5’ CTCAAGTTCAGCCACAGA 3’ |
|  |  | Reverse: 5’ AGATGCCAGGAGAATACC 3’ |
| shp | NM_011850 | Forward: 5’ ATCTCTTCTTCCGCCCTATC 3’ |
|  |  | Reverse: 5’ GTCACCTCAGCAAAAGCA 3’ |
| stat3 | NM_011486 | Forward: 5’ CACCTTGGATTGAGAGTCAAGAC 3’ |
|  |  | Reverse: 5’ AGGAATCGGCTATATTGCTGGT 3’ |
| sod1 |  | Forward: 5’ AAC CAG TTG TGT TGT CAG 3’ |
|  |  | Reverse: 5’ CAG CCT TGT GTA TTG TCC 3’ |
| sod2 |  | Forward: 5’ CAC GCT TAC TAC CTT CAG 3’ |
|  |  | Reverse: 5’ CAT TCT CCC AGT TGA TTA CA 3’ |
| gpx1 | NM_008160 | Forward: 5’ CCACCGTGTATGCCTTCTCC 3’ |
|  |  | Reverse: 5’ AGAGAGACGCGACATTCTCAAT 3’ |
| rpl4 |  | Forward: 5’ AGA AGA ATC CAC TGA AGA AC 3’ |
|  |  | Reverse: 5’ CTG GCG AAG AAT GGT ATT 3’ |
| β-actin | NM_007393.5 | Forward: 5’ GGC TGT ATT CCC CTC CAT CG 3’ |
|  |  | Reverse: 5’ CCA GTT GGT AAC AAT GCC ATG T 3’ |
| gapdh | GU214026.1 | Forward: 5’ TGC ACC ACC AAC TGC TTA G 3’ |
|  |  | Reverse: 5’ GGA TGC AGG GAT GAT GTT C 3’ |

Table S3: Reaction mixture for RT‒qPCR

| **Component** | **Volume/reaction** |
| --- | --- |
| Maxima SYBR Green qPCR Master Mix (2X) | 12.5 µL |
| Forward Primer | 0.3 µM |
| Reverse Primer | 0.3 µM |
| Template DNA | ≤ 500 ng |
| Water, nuclease-free | to 25 µL |
| Total Volume | 25 µL |

Table S4: Temperature and number of cycles of RT‒qPCR

| **Component** | **Temperature** | **Time** | **Number of Cycles** |
| --- | --- | --- | --- |
| Initial denaturation | 95 | 10 min | 1 |
| Denaturation | 95 | 15 s | 1 |
| Annealing/Extension | 60 | 60 s | 40 |

Table S5: Elution gradient of UHPLC

| Time (minute) | Mobile Phase A LC-grade Water + 0.1% formic acid | Mobile Phase B Acetonitrile (ACN) + 0.1% formic acid |
| --- | --- | --- |
| 1 | 95.5% | 0.5% |
| 15 | 95.5% - 0.5% | 0.5% - 95% |
| 4 | 0.5% | 99.5% |
| 2 | 0.5% - 95.5% | 99.5% - 0.5% |

Table S6: List of annotated metabolites in TRF group against HFD group. The fold-change (FC) as log^2^ and p values of the annotated metabolites are shown in the table as values against the HFD group.

| Metabolite | Fold-change (log2) | P value |
| --- | --- | --- |
| Isovaltrate | 7.6429 | 0.00149 |
| (+)-abscisic acid beta-D-glucopyranosyl ester | 5.9806 | 7.22E-05 |
| Paucin | 5.1866 | 4.45E-06 |
| 2,2-dichloro-1,1-ethanediol | 4.0956 | 0.045523 |
| Mupirocin | 3.8462 | 0.030777 |
| Taurochenodeoxycholic acid | 3.8371 | 0.019658 |
| L-Olivosyl-oleandolide | 3.7225 | 0.030254 |
| Taurohyocholic acid | 3.6062 | 0.019682 |
| Taurocholic acid | 3.2801 | 0.023508 |
| Ouabain | 2.9975 | 0.02805 |
| pseudaminic acid | 1.8028 | 0.006537 |
| (15Z)-9,12,13-Trihydroxy-15-octadecenoic acid | 1.5964 | 0.005335 |
| indospicine | 1.286 | 0.000424 |
| N-Acetyl-ala-ala-ala-methylester | 1.0483 | 0.009132 |
| 3-propylmalic acid | 0.86307 | 0.008511 |
| Palmitoyl sphingomyelin | 0.62613 | 0.041855 |
| Trigonelline | 0.59162 | 0.029791 |
| Glycerophosphocholine | -0.59431 | 0.029666 |
| 11-amino-undecanoic acid | -0.60037 | 0.0468 |
| Z-Arg-Arg-NHMec; Benzyloxycarbonylarginyl-arginine 4-methylcoumarin-7-ylamide | -0.62836 | 0.035491 |
| Artocarpin | -0.68462 | 0.008561 |
| lysophosphatidylethanolamine 0:0/20:4(8Z,11Z,14Z,17Z) | -0.70745 | 1.69E-05 |
| Biotin sulfone | -0.73369 | 0.029679 |
| C16 Sphingosine-1-phosphate | -0.74896 | 0.021534 |
| Glycerophospho-N-Oleoyl Ethanolamine | -0.81377 | 0.000621 |
| 1-octadecylglycero-3-phosphocholine | -0.82645 | 0.046111 |
| 4-Pyridoxic acid | -0.86159 | 0.007487 |
| Arachidonic acid | -0.87795 | 0.030662 |
| 2-Hydroxy-4-methylthiobutanoic acid | -0.88465 | 0.020117 |
| DL-Lysine | -0.88836 | 0.000675 |
| Allantoic acid | -0.89861 | 0.000472 |
| Istamycin C1 | -1.1599 | 0.010525 |
| D-Sphingosine | -1.3232 | 0.001413 |
| O-6-deoxy-a-L-galactopyranosyl-(1->3)-O-b-D-galactopyranosyl-(1->3)-O-2-(acetylamino)-2-deoxy-b-D-glucopyranosyl-[1->3(or 1->6)]-O-[O-b-D-galactopyranosyl-(1->4)-2-(acetylamino)-2-deoxy-b-D-glucopyranosyl-[1->6(or 1->3)]]-O-b-D-galactopyranosyl-(1->4)-D-G | -1.3292 | 0.016392 |
| Oleamide | -1.3343 | 0.038229 |
| Netilmicin | -1.3959 | 0.001809 |
| 3-Phenyllactic acid | -1.4653 | 0.011921 |
| N-(5Z,8Z,11Z,14Z-docosatetraenoyl)-ethanolamine | -1.4942 | 0.003432 |
| Hexadecanamide | -1.6835 | 0.025216 |
| C16 Sphinganine | -2.4489 | 9.82E-06 |
| 2-amino-hexadecanoic acid | -2.7841 | 2.10 x 10^-6^ |
| Phytosphingosine | -4.9224 | 6.48 x 10^-8^ |
| N-Undecanoylglycine | -6.1852 | 1.11 x 10^-9^ |

Table S7: List of biochemical pathways most likely modulated by TRF supplementation in mice fed a HFD. The matched features of the annotated genes and metabolites are based on code by the Kyoto Encyclopedia of Genes and Genomes (KEGG).

| Biochemical Pathway | Total Features | Hits | p value | log2 | Impact | Matched Features* |
| --- | --- | --- | --- | --- | --- | --- |
| Bile secretion | 246 | 5 | 0.0001 | 4.3893 | 0.11268 | mmu:20186; cpd:C05122; cpd:C05465; mmu:23957; cpd:C01443 |
| Biotin metabolism | 31 | 2 | 0.001179 | 2.9284 | 0.026316 | cpd:C00047; cpd:C20387 |
| Cholesterol metabolism | 59 | 2 | 0.00422 | 2.3747 | 0 | cpd:C05122; cpd:C05465 |
| Primary bile acid biosynthesis | 63 | 2 | 0.004798 | 2.319 | 0.021277 | cpd:C05465; cpd:C05122 |
| Longevity regulating pathway - multiple species | 64 | 2 | 0.004947 | 2.3056 | 0.09434 | mmu:20656; mmu:20655 |
| Sphingolipid metabolism | 73 | 2 | 0.006392 | 2.1944 | 0.13115 | cpd:C12144; cpd:C00319 |
| Peroxisome | 84 | 2 | 0.008387 | 2.0764 | 0 | mmu:20655; mmu:20656 |
| FoxO signaling pathway | 136 | 2 | 0.02098 | 1.6782 | 0.047619 | mmu:20656; mmu:20848 |
| Arachidonic acid metabolism | 164 | 2 | 0.029715 | 1.527 | 0.20513 | mmu:14775; cpd:C00219 |
| Taurine and hypotaurine metabolism | 33 | 1 | 0.053338 | 1.273 | 0.034483 | cpd:C05122 |
| Vitamin B6 metabolism | 37 | 1 | 0.059616 | 1.2246 | 0.025641 | cpd:C00847 |
| Regulation of lipolysis in adipocytes | 70 | 1 | 0.10992 | 0.95891 | 0.016667 | cpd:C00219 |

*mmu: *mus musculus* gene code; cpd: compound code
